# Does family support mediate the effect of anxiety and depression on maternal-fetal attachment in high-risk pregnant women admitted to the maternal-fetal intensive care unit?

**DOI:** 10.4069/kjwhn.2021.05.14

**Published:** 2021-06-24

**Authors:** Se-Hee Yoon, Mi-Hae Sung

**Affiliations:** 1 Busan Paik Hospital, Busan, Korea; 2 College of Nursing and Institute of Health Science, Inje University, Busan, Korea

**Keywords:** Anxiety, Depression, Hospitals, Pregnancy, Self-report

## Abstract

**Purpose:**

This study investigated the mediating effect of family support in the relationships of anxiety and depression with maternal-fetal attachment among pregnant women admitted to the maternal-fetal intensive care unit (MFICU) in Korea.

**Methods:**

The participants were high-risk pregnant women with a gestational age of at least 20 weeks who were admitted to MFICUs in Busan and Yangsan. The Korean versions of four measurement tools were used for the self-report questionnaire: Spielberger’s State-Trait Anxiety Inventory, the Edinburgh Postnatal Depression Scale, Cobb’s family support measurement, and Cranley’s maternal-fetal attachment scale. Data were collected from June 22 to September 20, 2020. Out of 124 participants, data from 123 respondents were analyzed. Descriptive statistics and regression analysis were done.

**Results:**

The average age of participants was 34.1 years. Their anxiety level was moderate (43.57±11.65 points out of 80) and 53.6% were identified as having moderate depression (average 10.13±5.48 points out of 30). Family support was somewhat high (average 43.30±5.03 points out of 55). The average score of maternal-fetal attachment was also somewhat high (73.37±12.14 points out of 96). Family support had a partial mediating effect in the relationships of anxiety and depression with maternal-fetal attachment among high-risk pregnant women admitted to the MFICU.

**Conclusion:**

Maintaining family support is challenging due to the nature of the MFICU. Considering the mediating effect of family support, establishing an intervention plan to strengthen family support can be helpful as a way to improve maternal-fetal attachment for high-risk pregnant women admitted to the MFICU.

## Introduction

Due to the recent increase in women’s social status and activity in the workforce, the proportion of older and high-risk pregnant women over 35 is also increasing. The number of pregnant women who received hospitalization or outpatient care for high-risk pregnancies in South Korea increased from 27,223 in 2009 to 142,565 in 2019, corresponding to approximately six-fold growth over 10 years [1]. High-risk pregnancies cause high-risk deliveries of high-risk newborn babies [2], and constitute a social problem that increases medical costs, reduces population health [3], and increases individual and national economic losses [4]. Against this background, the South Korean government established a policy to support the establishment of maternal neonatal intensive care centers as a public health policy project. This policy provides systematic therapeutic management and nursing for high-risk pregnant women in the maternal-fetal intensive care unit (MFICU), and as of 2021, 19 hospitals are operating MFICUs. MFICUs provide intensive management, including fetal monitoring for pregnant women with gestational hypertension, gestational diabetes, heart disease, and complications such as postpartum bleeding and sepsis [5]. Fetal attachment refers to the development of emotional bonds with the fetus, prompting women to participate in interactive behaviors with and show affection for the fetus [6]. It is an important factor in helping pregnant women develop and adapt during pregnancy and influences healthy fetal development [7].

Pregnant women can experience depression due to changes in hormone levels and emotions related to pregnancy [8]. Depression has negative consequences for childbirth, such as preterm delivery, low birth weight, and difficulty forming bonds with the baby after childbirth [9]. Pregnant women admitted to the MFICU often experience panic and frustration due to the sudden diagnosis of a high-risk pregnancy, uncertain prognosis, and hospitalization [4]; thus, they often experience higher anxiety than women with normal pregnancies [10]. Depressed pregnant women have less interest in the fetus and interact less with the fetus, making it challenging to form maternal-fetal attachment [9]. Previous studies of maternal-fetal attachment have shown that the higher anxiety and depression of hospitalized high-risk pregnant women negatively affect fetal attachment, whereas family support reduces these adverse effects [2,9,11].

With the recent increase in MFICUs across Korea and the restrictive nature of the MFICU setting, multiple issues are foreseeable for high-risk pregnant women. However, insufficient research has explored factors associated with fetal attachment and the mediating effects of family support, both in domestic and international contexts. No studies have been conducted on the effects of anxiety and depression on fetal attachment and the mediating effects of family support in that relationship among high-risk pregnant women admitted to the MFICU.

This study aimed to identify factors affecting maternal-fetal attachment and the mediating effect of family support among high-risk pregnant women admitted to MFICU in Korea. The specific aims were (1) to identify the factors affecting the participants’ maternal-fetal attachment, and (2) to identify the mediating effect of family support on mater­nal-fetal attachment.

## Methods

Ethics statement: This study was approved by the Institutional Review Board of Inje University (2020-04-022-003). Informed consent was obtained from the participants.

This is a self-report-based study using a correlational survey design. This manuscript was written in accordance with the Strengthening the Reporting of Observational Studies in Epidemiology (STROBE) guidelines (https://www.strobe-statement.org/index.php?id=strobe-home).

### Sample and sampling

The participants of this study were women who were diagnosed with high-risk pregnancy and admitted to the MFICU of Inje University Busan Paik Hospital in Busan and Pusan National University Yangsan Hospital in Yangsan. Pregnant women who fulfilled the following four criteria were included via convenience sampling: (1) admission after being diagnosed with premature labor, early amniotic membrane rupture, cervical incompetence, placenta previa, premature rupture of amniotic membrane, preeclampsia, oligohydramnios, abruptio placentae, uterine malformation, multiple pregnancies, polyhydramnios, fetal abnormalities (fetal growth delay, fetal malformation, or fetal lyrics), and relevant medical conditions (high blood pressure, diabetes, autoimmune diseases, blood clotting disorders, etc.); (2) at least 20 weeks of pregnancy; (3) a hospital stay of more than 3 days; and (4) comprehension of the purpose of the study and voluntary agreement to participate. The following two exclusion criteria were applied: (1) diagnosis of psychiatric diseases such as depression and cognitive dysfunction; and (2) unstable conditions that could affect the mother or fetus, such as delivery in progress or active bleeding. There was no identifiable bias in the selection or exclusion process.

**Figure 1.**
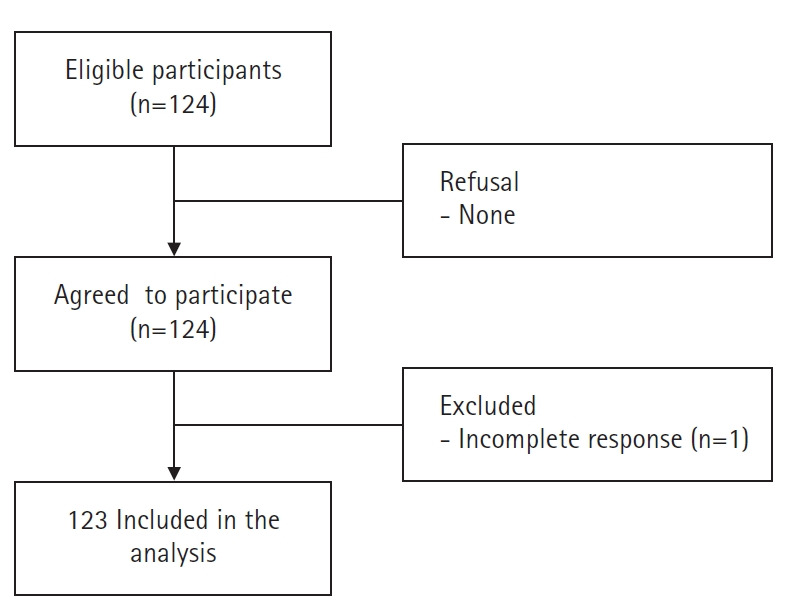
Flow diagram of recruitment

The sample size required for this study was calculated using G*Power version 3.1.9.4. Based on previous studies [12], the following conditions were entered: effect size, .15 (medium size); significance level, .05; power, .80; and seven predictor variables (age, religion, high-risk pregnancy experience, planned pregnancy, anxiety, depression, and family support). The minimum number of samples required for multiple regression analysis was calculated as 103 participants, and a dropout rate of 20% was anticipated based on a previous study [12]. All eligible women were invited to take part in the study; out of a total of 124 participants, all responded to the questionnaire. Excluding one inadequate response, 123 participants were included in the analysis (Figure 1).

### Instruments

The original developers and Korean translators gave permission to use all the measurement tools used in this study. Measurement tools for anxiety, depression, family support, and maternal-fetal attachment were used. The time to complete the paper questionnaire was about 10 minutes.

#### Maternal-fetal attachment

Cranley’s maternal-fetal attachment scale (MFAS) was developed for pregnant women [6], and the Korean version was developed by Kim [13]. This tool consists of a total of 24 items; three items that distinguish oneself from the fetus, six items that speculate on the characteristics and intentions of the fetus, four items related to role acceptance, five items that ask about interaction with the fetus and the woman herself, and six items related to commitment. The responses are given on a 4-point Likert scale (1, definitely no; 4, definitely yes). The scores range from 24 to 96 points, and higher scores indicate a greater degree of maternal-fetal attachment. At the time of development, Cronbach’s α of the original tool was .85 [6]. Internal reliability was good for the translated tool (Cronbach’s α=92) [13] and in the present study (Cronbach’s α=.92).

#### Family support

Cobb’s family support measurement tool was developed for patients with various pathologies [14] and translated into Korean by Kang [15]. This 11-item tool measures the degree of family support perceived by the patient with a 5-point Likert scale (1, not at all; 5, very much). The total score ranges from 11 to 55 points, and higher scores indicate a higher degree of family support. The Cronbach’s α value of the tool at the time of development was .89 [14], and it was .86 for the translated tool [15]. Internal reliability was good in the present study (Cronbach’s α=.87).

#### Anxiety

The Korean version of Spielberger’s State-Trait Anxiety Inventory [16], translated by Kim and Shin [17], was developed for measuring anxiety in normal adults without mental disorders. In this study, the 20 items on present state anxiety were included to measure anxiety levels in hospitalization. The summed scores using the 4-point Likert scale (1, not at all; 4, always) yielded a possible range from 20 to 80 points, with higher scores corresponding to a higher degree of anxiety. Cronbach’s α was .84 at the time of development [16], .87 for the Korean translation [17], and .94 in the present study.

#### Depression

The Edinburgh Postnatal Depression Scale (EPDS) was initially developed to screen for postpartum depression [18] and has also been used in pregnancy [19]. The Korean translation by Han et al. [20] was used. It measures respondents’ mood for the last week with 10 items on a 4-point Likert scale (0, no, never; 3, yes, most of the time; or variable descriptions), and positive items are handled by inverse conversion. The total score ranged from 0 to 30, and a higher score indicates more severe depression. Those with a score of 10 or higher are classified as requiring psychiatric evaluation in Korea [20], with scores of 10 to 12 points classified as mild depression and a score of more than 12 requiring careful attention for major depressive disorder. Cronbach’s α was .87 for the original tool [18], .85 for the translated tool [20], and .84 in the present study.

#### General characteristics and obstetric characteristics

The general characteristics consisted of eight items, including current age, marital status, marital period, education, occupation, monthly income, religion, and the number of family members. The obstetric characteristics consisted of 15 items, including the number of days of hospitalization, diagnosis, and whether the pregnancy was planned. These items were designed by the authors according to the literature.

### Procedures

The survey data were collected from June 22 to September 20, 2020. Questionnaires were distributed by the research team and collected after completion on the same day. A gift for the newborn (worth 2 US dollars [USD]) was provided as a token of appreciation.

### Statistical methods

**Figure 2.**
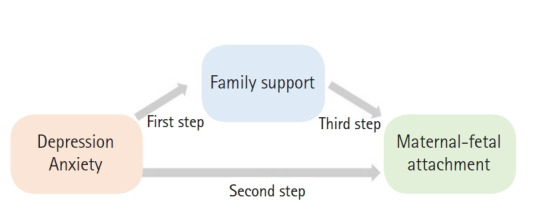
Relationships among the two independent variables (depression and anxiety), the mediator (family support), and dependent variable (maternal-fetal attachment) and the analysis steps.

Skewness and kurtosis were tested to confirm whether the data had a normal distribution. The general characteristics, obstetric characteristics, anxiety, depression, and degree of maternal-fetal attachment were analyzed using descriptive statistics, and factors affecting maternal-fetal attachment were analyzed using hierarchical multiple regression. The procedure proposed by Baron and Kenny [21] was used to confirm the mediating effect of family support, and the Sobel test was performed to verify the effectiveness of the mediating effect path. The independent variables were anxiety and depression, which showed significant correlations with fetal attachment. Family support was introduced as a mediator. In the first step, it was checked whether the independent variables (anxiety and depression) affected the mediator (family support). In the second step, it was checked whether the independent variables (anxiety and depression) had a significant effect on the dependent variable (fetal attachment). In the final step, it was checked whether the independent variables (anxiety and depression) and the mediator (family support) had a significant effect on the dependent variable (fetal attachment). The mediating effect was confirmed by comparing the regression coefficient in step 2 with the regression coefficient in step 3. The analytical process is presented in Figure 2. The collected data were analyzed using IBM SPSS ver. 25.0 (IBM Corp., Armonk, NY, USA).

## Results

### General and obstetric characteristics of the participants

The average age of the participants was 34.1 years old, and 53.7% were under 35 years old. The average length of marriage was 46.63 months, and 39.1% of participants had been married for less than 24 months. The proportion of participants with a spouse was 96.7%, and 78.9% of the participants had a terminal education level of college graduation. Furthermore, 51.2% of participants were employed. The average monthly household income was 5.22 million Korean won (USD 4,626), and 49.6% of the group had monthly household incomes between 3 million Korean won (USD 2,659) and 6 million Korean won (USD 5,318). The majority of the participants (69.1%) were not religious, and 61.8% lived with their husbands (Table 1).

The average gestational age was 32.08 weeks, and 39.8% of the participants’ gestational age was between 28 weeks and 37 weeks. Fewer than half of the women (38.2%) were pregnant for the first time, while 31.7% previously had a natural abortion, and 7.3% previously had an artificial abortion. In addition, 13.8% of the participants had a history of preterm birth, and 28.5% of the participants had a previous full-term delivery. The proportion of the participants who had previously experienced a high-risk pregnancy was 30.1%. Most of the participants (61.0%) had spontaneous natural pregnancies and planned pregnancies (65.0%). The average length of hospitalization was 5.02 days, and 72.4% were hospitalized for 3 days. Multiple diseases were diagnosed in 56.3% of the participants. The proportion of participants with medical illnesses was 40.7%, while 35.8% of participants had preterm labor and 26.8% had fetal abnormalities. The other obstetric conditions were as follows; cervical incompetence (21.1%), multiple pregnancies (19.5%), placenta previa (13.8%), premature rupture of amniotic membrane (11.4%), preeclampsia (9.8%), uterine malformation (8.9%), oligohydramnios (4.9%), abruptio placentae (1.6%), and polyhydramnios (0.8%) (Table 2).

### Participants’ anxiety, depression, family support, and fetal attachment

The participants’ anxiety score was medium (43.57±11.65 points out of a maximum of 80). For depression, the average was 10.13±5.48 points out of a maximum of 30, with 66 (53.6%) identified as likely depressed (score of 10 or more) and 42 women (34.1%) having levels highly suspicious for depression (score of 13 or greater). The average score of family support was somewhat high (43.30±5.03 points out of a maximum of 55). The average score of maternal-fetal attachment was also somewhat high (73.37±12.14 points out of a maximum of 96) (Table 3).

### Mediating effects of family support in the relationships of anxiety and depression with maternal-fetal attachment

We examined the autocorrelation of the dependent variable and the multicollinearity between the independent variables before verifying the mediating effect. The Durbin-Watson statistic yielded results of 2.10 to 2.45, which were close to 2. Therefore, the assumption of the independence of the residuals could be accepted. Tolerance ranged from .74 to .95, all above .10, and the variance inflation factor ranged from 1.04 to 1.33, all below 10, indicating no multicollinearity.

Controlling for age, which was a significant factor, in the first step, it was found that anxiety, an independent variable, had a significant effect on the mediator (family support) (β=–.50, *p*<.001). In the second step, anxiety was found to have a significant effect on maternal-fetal attachment (dependent variable) (β=–.55, *p*<.001). In the third step, anxiety (independent variable) and family support (mediator) had significant effects on maternal-fetal attachment (dependent variable) (anxiety: β=–.42, *p*<.001; family support: β=.25, *p*= .002). In the third step, the explanatory power of anxiety and family support was 41.9%. The β value of anxiety in the third step was –.42, the absolute value of which was less than that in the second stage, –.55, indicating a mediating effect of family support. Since both the independent variable and the mediator significantly affected the dependent variable, it was found that family support partially mediated the effect of anxiety on maternal-fetal attachment. Furthermore, the Sobel test showed the significance of the mediating effect (z=–2.87, *p*=.003).

Controlling for age, which was a significant factor, depression, an independent variable, had a significant effect on the mediator (family support) in the first step (β=–.45, *p*<.001). In the second step, depression was found to have a significant effect on maternal-fetal attachment (dependent variable) (β=–.56, *p*<.001). In the third step, depression (β=–.44, *p*<.001) and family support (β=.26, *p*=.001) were found to have statistically significant effects, and the explanatory power of depression and family support was 43.5%. The β value of depression in the third step was –.44, the absolute value of which was lower than the β-value in the second step, –.56. Therefore, it can be concluded that family support mediated the effect of depression on maternal-fetal attachment. Since both the independent variable and the mediator significantly affected the dependent variable, it can be concluded that family support partially mediated the effect of depression on fetal attachment. Furthermore, according to the Sobel test, the mediating effect (z=–3.03, *p*=.002) was significant (Table 4).

## Discussion

The factors influencing maternal-fetal attachment in high-risk pregnant women admitted to MFICU were depression, anxiety, and family support, of which depression was the most influential factor. Conducting screening for prenatal depression using an available tool would make it possible to effectively screen and manage patients with significant depression within a short time at a low cost. Systematic and ongoing management of prenatal depression may help not only reduce patients’ depression but also improve maternal-fetal attachment. According to a prior study [22], support through collective counseling helps reduce depression and enhances hope and optimism. Thus, it may be helpful to provide supportive counseling for high-risk women admitted to MFICU. Other influencing factors were anxiety and family support. This result is similar to the findings of a previous study in high-risk pregnant women [2] that reported that anxiety and marital adaptation influenced maternal-fetal attachment. Another study of women who experienced miscarriage noted that spousal support was a factor associated with maternal-fetal attachment [12].

The partial mediating effect of family support was confirmed in the relationships of anxiety and depression with maternal-fetal attachment in high-risk pregnant women admitted to MFICU. High anxiety and depression levels may reduce family support, which can negatively affect maternal-fetal attachment. Family support acts as a mediator to reduce the decline in maternal-fetal attachment caused by anxiety and depression. Although family members should be encouraged to visit the hospital during permitted hours to support high-risk pregnant women, their ability to do is very limited in the MFICU due to the nature of this setting and the constant surveillance required. Although the data collection period was during the COVID-19 pandemic, the limited opportunity for in-person support from family is inherent to the MFICU. Family support via smartphones is an alternative, but more structured “visiting” modes that encourage engagement and supportive interactions are needed.

The participants in this study reported relatively high maternal-fetal attachment scores, with an average of 73.37 out of 96 points, which is similar to the result of 74.25 points in a study of high-risk pregnant women [10] using the same tool. However, it is lower than the average of 81.36 points in a study of women with normal pregnancies women [7]. These results imply that high-risk pregnant women admitted to the MFICU have a lower degree of maternal-fetal attachment than women with normal pregnancies (i.e., without pregnancy complications or other diseases).

In this study, the average anxiety score was 43.57 out of a total of 80. This is higher than previously reported scores of 39.02 points in high-risk pregnancies [2] and 39.3 points in normal pregnancies [9] using the same tool. As state anxiety ranged from 21 to 79 points, reflecting significant variation among individuals, nursing interventions for pregnant women in the MFICU should carefully note differences in individual characteristics and the degree of anxiety.

The average depression score (10.13 out of a total of 30) was also higher than previously reported among pregnant women (7.8 points) [23]. In this study, 53.6% of participants required a psychiatric evaluation (score≥10). Furthermore, 34.1% were found to be at risk for severe depression (score≥13), which is higher than the proportion of 20.6% reported for generally healthy pregnant women [23]. Underlying diseases, early labor pains, and fetal abnormalities were present in a large proportion of participants in this study, which is believed to have affected depression among pregnant women.

Although it is difficult to compare the findings directly due to insufficient research using the same tool among high-risk pregnant women, the degree of family support in this study (43.3 out of 55) appears to be similar to that reported for women with normal pregnancies (17.84 out of 20) using the multidimensional scale of perceived social support tool [24]. There is a need for further research on family support among high-risk pregnant women admitted to the MFICU.

Since the participants of the study were selected through convenience sampling from two hospitals in one region, caution is needed when generalizing the results to other institutions in other regions or countries. Nonetheless, this study is meaningful in that it examined high-risk women in the MFICU; as such, future studies would be beneficial to expand the target participants through nationwide sampling. It is also necessary to conduct further studies to develop nursing intervention programs that can strengthen family support and engagement with high-risk pregnant women during their stay in the MFICU to improve maternal-fetal attachment.

In conclusion, this study found that lower levels of anxiety and depression, as well as higher levels of family support, were associated with higher maternal-fetal attachment. Depression was identified as the most influential factor, and the mediating effect of family support in the relationships of depression and anxiety with maternal-fetal attachment was verified. Therefore, it is necessary to reduce negative emotions such as anxiety and depression, and active family support is necessary to overcome those negative emotions. Developing a nursing intervention program to reduce anxiety would also support pregnant women and their family. It is necessary to diagnose depression early through a prenatal depression screening system to enable early interventions. It would also be worth considering preparing an educational program to cultivate professional counselors for high-risk pregnant women with formal certificates for those who complete the course.

## Figures and Tables

**Table 1.** General characteristics of participants (N=123)

Characteristic	Categories	n (%)	Mean±SD	t/F	*p*
Age (year)	<35	66 (53.7)	76.46±10.94	3.15	.002
	≥35	57 (46.3)	69.78±12.56		
Spouse	Yes	119 (96.7)	73.61±11.62	0.59	.595
	No	4 (3.3)	66.20±24.77		
Length of marriage (month)	≤24	48 (39.0)	75.81±13.07	2.84	.062
	25–72	59 (48.0)	70.69±10.78		
	≥73	16 (13.0)	75.93±12.66		
Education	≤High school	15 (12.2)	71.73±9.17	2.66	.073
	University (college)	97 (78.9)	72.73±12.15		
	≥Graduate school	11 (8.9)	81.27±13.68		
Job	Yes	63 (51.2)	72.82±13.34	–0.51	.610
	No	60 (48.8)	73.95±10.83		
Monthly household income (KRW)	≤300 million	30 (24.4)	71.13±12.85	0.81	.444
	3.01-6 million	61 (49.6)	73.60±11.60		
	≥6.01 million	32 (26.0)	75.01±12.55		
Religion	Yes	38 (30.9)	72.94±11.25	–0.25	.796
	No	85 (69.1)	73.56±12.58		
Cohabiting family	Living without spouse	6 (4.9)	68.00±20.48	0.77	.462
	Living with spouse only	76 (61.8)	74.11±11.91		
	Living with spouse & others	41 (33.3)	72.78±11.20		

KRW: Korean won (1 million KRW is approximately 900 US dollars).

**Table 2.** Obstetric characteristics of participants (N=123)

Characteristic	Categories	n (%)	Mean±SD	t/F	*p*
Gestational period (week)	≤27	35 (28.5)	71.94±10.22	1.37	.256
	28–36	49 (39.8)	72.28±13.19		
	≥37	39 (31.7)	76.02±12.23		
Experience of spontaneous abortion	Yes	39 (31.7)	71.41±10.88	–1.22	.223
	No	84 (68.3)	74.28±12.64		
Number of pregnancies	1	47 (38.2)	74.89±13.62	0.60	.550
	2	42 (34.1)	72.26±10.61		
	≥3	34 (27.6)	72.64±11.89		
Experience of induced abortion	Yes	9 (7.3)	75.66±8.58	0.58	.559
	No	114 (92.7)	73.19±12.39		
Experience of preterm delivery	Yes	17 (13.8)	68.52±12.28	–1.78	.076
	No	106 (86.2)	74.15±11.99		
Experience of full-term delivery	Yes	35 (28.5)	74.17±11.04	0.45	.648
	No	88 (71.5)	73.05±12.60		
Experience of high-risk pregnancy	Yes	37 (30.1)	70.89±12.36	–1.49	.138
	No	86 (69.9)	74.44±11.96		
Pregnancy method	Natural pregnancy	75 (61.0)	73.52±12.16	0.02	.995
	Ovulation induction	5 (4.1)	74.20±16.78		
	Intrauterine insemination	6 (4.9)	72.66±11.44		
	*In vitro*fertilization	37 (30.1)	73.08±12.07		
Planned pregnancy	Yes	80 (65.0)	73.70±11.88	0.40	.686
	No	43 (35.0)	72.76±12.74		
Hospitalization days	3	89 (72.4)	73.97±12.54	1.01	.365
	4–5	15 (12.2)	74.40±10.86		
	≥6	19 (15.4)	69.73±11.02		
Number of diagnosed diseases	1	54 (43.9)	72.70±11.78	0.79	.454
	2	39 (31.7)	72.43±11.88		
	≥3	30 (24.4)	75.80±13.17		
Disease	Preterm labor	44 (35.8)			
	Premature rupture of membrane	14 (11.4)			
	Incompetent internal orifice of cervix	26 (21.1)			
	Placenta previa	17 (13.8)			
	Placenta abruptio	2 (1.6)			
	Preeclampsia	12 (9.8)			
	Polyhydramnios	1 (0.8)			
	Oligohydramnios	6 (4.9)			
	Uterus anomaly	11 (8.9)			
	Multifetal gestation	24 (19.5)			
	Fetal abnormality	33 (26.8)			
	Maternal disease	50 (40.7)			

**Table 3.** Degree of anxiety, depression, family support, and maternal-fetal attachment (N=123)

Variable	n (%)	Score		
		Possible range	Mean±SD	Range
Anxiety		20–80	43.57±11.65	21–79
Depression		0–30	10.13±5.48	0–22
Normal	57 (46.3)			
Likely depressed	24 (19.5)			
Major depression	42 (34.1)			
Family support		11–55	43.30±5.03	30–50
Maternal-fetal attachment		24–96	73.37±12.14	36–96

**Table 4.** The mediating effect of family support on the relationships of anxiety and depression with maternal-fetal attachment (N=123)

Variable		B	SE	β	t	*p*	Adjusted R²	F
1	Anxiety → Family support	–0.21	.03	–.50	–6.39	<.001	.246	40.82
2	Anxiety → Maternal-fetal attachment	–0.57	.07	–.55	–7.68	<.001	.376	37.79
3	Anxiety → Maternal-fetal attachment	–0.44	.08	–.42	–5.35	<.001	.419	30.38
	Family support → Maternal-fetal attachment	0.60	.19	.25	3.15	.002		
	Sobel test: Z=–2.95, *p*=.003							
1	Depression → Family support	–0.41	.07	–.45	–5.58	<.001	.198	31.17
2	Depression → Maternal-fetal attachment	–1.25	.16	–.56	–7.77	<.001	.381	38.70
3	Depression → Maternal-fetal attachment	–0.09	.17	–.44	–5.72	<.001	.435	32.34
	Family support → Maternal-fetal attachment	0.64	.18	.26	3.52	.001		
	Sobel test: Z=–3.07, *p*=.002							

Age is adjusted.
